# Introduction to *Pristionchus pacificus* anatomy

**DOI:** 10.21307/jofnem-2021-091

**Published:** 2021-11-03

**Authors:** Nathan E. Schroeder

**Affiliations:** 1 Department of Crop Sciences, University of Illinois at Urbana-Champaign, Urbana, IL, 61801

**Keywords:** Cytology, Electron microscopy, Free-living nematode, Morphology, Ultrastructure, WormAtlas

## Abstract

*Pristionchus pacificus* has emerged as an important nematode species used to understand the evolution of development and behavior. While *P. pacificus* (Diplogasteridae) is only distantly related to *Caenorhabditis elegans* (Rhabditidae), both use an identical reproductive strategy, are easily reared on bacteria in Petri dishes and complete their life cycles within a few days. Over the past 25 years, several detailed light and electron microscopy studies have elucidated the anatomy of *P. pacificus* and have demonstrated clear homology to many cells in *C. elegans*. Despite this similarity, sufficient anatomical differences between *C. elegans* and *P. pacificus* have allowed the latter to be used in comparative evo-devo studies. For example, the stoma of *P. pacificus* contains a large dorsal tooth used during predation on other nematodes when supplementing its primarily bacterial diet. This review discusses the main anatomical features of *P. pacificus* with emphasis on comparison to *C. elegans.*

In 1988, the nematode *Pristionchus pacificus* was isolated from garden soil in Pasadena, CA (Sommer et al., 1996). *P. pacificus* was originally studied as a comparative species to *Caenorhabditis elegans* (Fig. 1). Both species are easily reared in the laboratory on bacterial lawns and have similar anatomies and life cycles (Hong and Sommer, 2006). *P. pacificus* uses a facultative hermaphroditic reproductive strategy similar to *C. elegans.* Many of the laboratory tools developed for *C. elegans* research are readily transferable to *P. pacificus* (Pires da Silva, 2013). The amenability of *P. pacificus* to genetic manipulation and analysis has propelled this species forward as an important comparative model for understanding the molecular basis of evolution for diverse traits.

**Figure 1:**
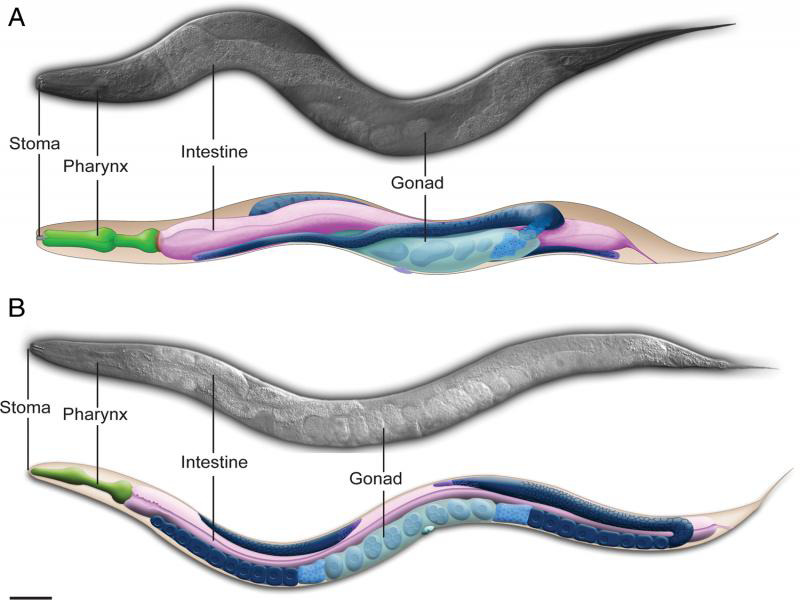
*Pristionchus pacificus* (A) and *Caenorhabditis elegans* (B) adult hermaphrodites have similar sizes and shapes. Scale bar 50 µm.

Hundreds of *P. pacificus* isolates and other related *Pristionchus* species from around the world have been isolated (McGaughran and Morgan, 2015). Due to its cosmopolitan distribution and the diversity of isolated populations, *P. pacificus* has emerged as a powerful model for studying evolutionary processes at the molecular level. Many *P. pacificus* isolates are found associated with beetles and data suggests a necromenic relationship wherein the nematode waits for the beetle to die and then feeds off of the rotting cadaver and associated microorganisms (Herrmann et al., 2006). However, others have proposed that the relationship between *P. pacificus* and beetles is phoretic – the nematodes use beetles as a means of transport to a new nutrient rich environment (Félix et al., 2018).

*P. pacificus* is androdioecious, comprising both self-fertile hermaphrodites (XX) and occasional males (XO) that fertilize hermaphrodites. Males are usually less than 1% of the population; however, this fraction varies among *P. pacificus* isolates and environmental conditions (Morgan et al., 2017). The androdioecious system is valuable for genetic studies as selfing by hermaphrodites leads to genetically identical offspring, while the presence of males allows for crosses between distinct genotypes.

## Life cycle

**Figure 2:**
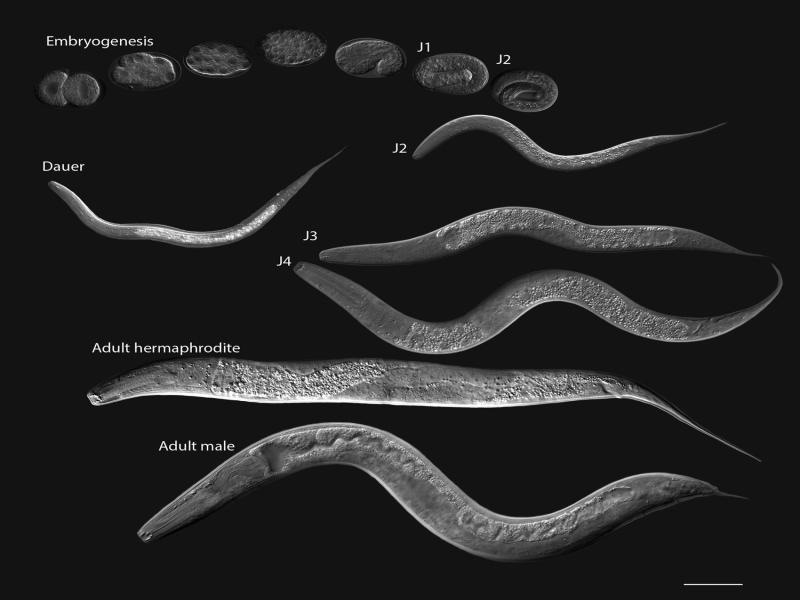
Life cycle of *Pristionchus pacificus*. Differential interference contrast images of developmental stages. Development from the two-cell stage to adult takes approximately 72 hrs at 20°C. Each post-embryonic stage is positioned with its left side facing the reader, except the J4, which is facing the right side. Scale bar, 50 µm.

*P. pacificus* is easily maintained on bacterial cultures in the laboratory and frozen stocks can be kept for years (Pires da Silva, 2013). Development from fertilized egg to adult is completed in approximately four days which is only slightly longer than *C. elegans* (Fig. 2) (Hong and Sommer, 2006). Following exposure to harsh environmental conditions, *P. pacificus* can form a stress-resistant dauer stage that can survive extended periods without feeding.

**Figure 3:**
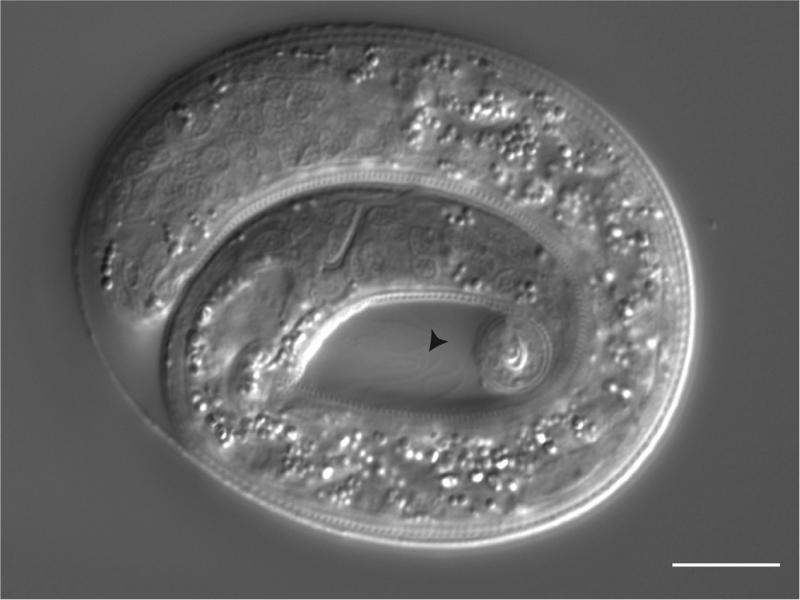
Unhatched J2. The presence of a shed J1 cuticle (arrowhead) is indicative of the J1 to J2 molt occurring prior to hatching. Scale bar, 10 µm.

Unlike *C. elegans,* which hatches as a J1, *P. pacificus* does not emerge from its egg until J2 (Fig. 2). The presence of a distinct J1 stage in *P. pacificus* is marked by ecdysis (shedding of the cuticle) within the egg-shell (Fürst von Lieven, 2005) (Fig. 3). The shift in timing of *P. pacificus* hatch may allow for the development of moveable teeth within the stoma. A similar delay is seen in many plant-parasitic nematodes with moveable stylet mouthparts. Despite the delay in hatching, the generation time for *P. pacificus* is only slightly longer than *C. elegans* (Hong and Sommer, 2006). Some events that occur in the post-hatch J1 of *C. elegans* occur prior to hatching in J1 *P. pacificus.* For example, migration of the Pn ectoblasts, which contribute to the post-embryonic development of the ventral nerve cord and vulva, occurs in the J1 of both species (Félix et al., 1999; Sulston and Horvitz, 1977). Unfortunately, the additional post-embryonic development and corresponding movement within the eggshell will make it challenging to describe the complete embryonic cell lineage of *P. pacificus.*

## Cuticle

**Figure 4:**
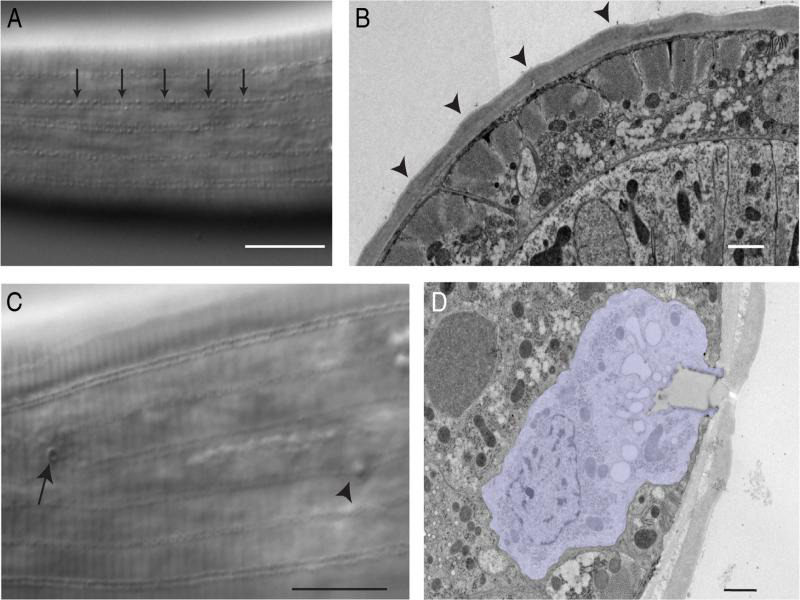
(A) The cuticle of *P. pacificus* contains longitudinal stippled lines around the circumference of the animal. Scale bar, 10 µm. (B) In an EM cross section, this stippling can be seen as waves around the animal (arrowheads). Scale bar, 1 µm. Image source: Ralf Sommer Lab, Bumbarger13–1301. (C, D) The cuticle of *P. pacificus* is interrupted by epithelial gland cell pores. In DIC (C), the small gland cell pores (arrow) are occasionally visible both dorsal and ventral of the midline (deirid, arrowhead). Scale bar, 10 µm. In EM cross sections (D), the epithelial gland cell (purple) is surrounded by the hypodermis. Scale bar, 1 µm. Image Source: Ralf Sommer, Bumbarger14–1955.

The cuticle of *P. pacificus* is a tough rigid extracellular matrix likely secreted by the underlying epithelial cells. Similar to *C. elegans* and other nematodes, collagen is a major biochemical component of the body wall (Kenning et al., 2004; Schlager et al., 2009). The cuticle of *P. pacificus* contains evenly spaced stippled longitudinal ridges along the length of the body (Fig. 4). Unlike the *C. elegans* alae, these longitudinal ridges are not restricted to the lateral field of the cuticle. Several tissues open to the outside through the cuticle including the stoma and anus. The excretory pore of *P. pacificus* lies on the ventral ridge near the terminal bulb of the pharynx. The lateral cuticle of *P. pacificus* hermaphrodites is also interrupted by a series of small pores that open to gland cells embedded within the lateral hypodermis (Fig. 4) (Ragsdale et al., 2015; Sommer et al., 1996). These lateral gland cells are not present in *C. elegans* and their lineage in *P. pacificus* is unknown.

*P. pacificus* dauers tend to retain their L2 cuticle for extended periods of time. Retention of the L2 cuticle likely assists dauers in survival of adverse environmental conditions. *P. pacificus* dauers also secrete a waxy ester onto the cuticle surface that facilitates aggregation of dauers and the formation of ‘dauer towers’, which enhance the nematode’s ability to attach to a beetle host (Penkov et al., 2014).

## The epithelial system

The main epithelial system of *P. pacificus* consists of hypodermal syncytia. Similar to *C. elegans,* the hypodermis wraps around the body wall of the nematode alternating between thick regions with nuclei in the cords and a thin sheet-like region underlying the somatic muscles. The hypodermis is interrupted on the lateral ridges by a linear set of 15 epithelial ‘seam’ cells per side in the adult hermaphrodite, which is slightly less than the 16 homologous seam cells found in *C. elegans* (Cinkornpumin et al., 2014). Also, differing from *C. elegans,* the *P. pacificus* seam cells send processes from the apical membrane that extend away from the lateral midline.

## The nervous system

As with *C. elegans* and other nematodes, the majority of *P. pacificus* neurons are located in the head and tail. The anterior nervous system of *P. pacificus*, including the pharynx and anterior amphid sensory neurons, have been reconstructed from serial-section electron microscopy (Bumbarger et al., 2013; Hong et al., 2019). The nervous system of *P. pacificus* is remarkably similar to that of *C. elegans.* Both the anterior amphid sensory sensilla and the pharyngeal nervous system have equivalent numbers of neurons in both species and many of these are obvious homologs based on their positions and structures (Bumbarger et al., 2013; Bumbarger and Riebesell, 2015; Hong et al., 2019). While both species have 12 pairs of amphid neurons, the sensory ending shape differs between some homologous neurons. For example, the *P. pacificus* amphids lack obvious wing-like ciliated dendrites found in the AWA, AWB, and AWC neurons in *C. elegans* (Hong et al., 2019).

While the pharyngeal nervous system of both species comprises 20 neurons, the synaptic connectivity differs between these species (Bumbarger et al., 2013), although a more recent reanalysis of the *C. elegans* pharyngeal connectome suggests these differences may not be as great as originally reported (Cook et al., 2020). Wiring differences between the two species in the amphid and pharyngeal circuits may mediate behavioral differences (Bumbarger et al., 2013; Hong et al., 2019). Similarly, slight differences in neurotransmitter expression occur between *P. pacificus* and *C. elegans* neurons (Loer and Rivard, 2007). While there are differences reported in the number of neurons within the ventral nerve cord between *C. elegans* and *P. pacificus,* additional electron microscopy will be needed to determine the extent of neuronal differences between these species (Han et al., 2016).

## The muscle system

**Figure 5:**
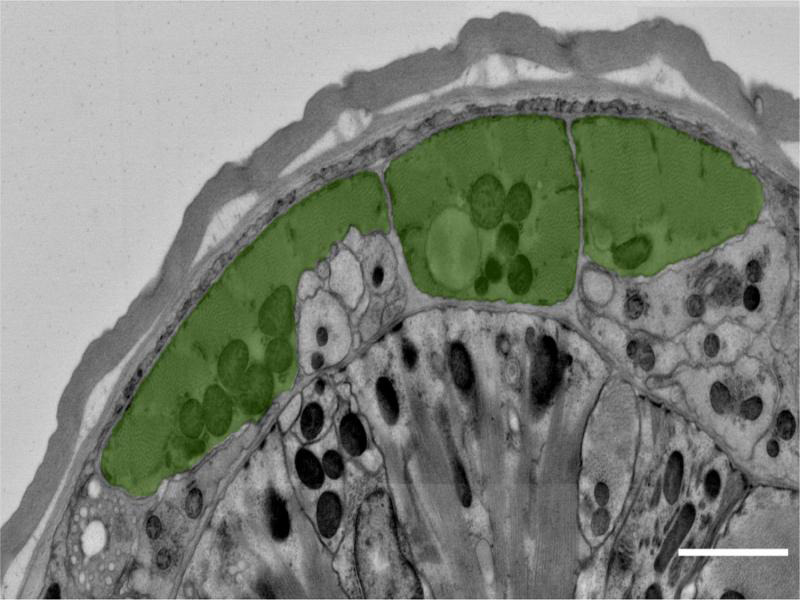
Transverse EM section through the *P. pacificus* head showing body wall muscles (green) lying against body wall. Scale bar, 1 µm.

The somatic body wall muscle of *P. pacificus* is platymyarian and similar in overall structure to *C. elegans* (Fig. 5). A basal lamina separates neurons and hypodermis from the body wall muscle. Typical to all nematodes, innervation of *P. pacificus* body wall muscles occurs through muscle arm processes extending to neurons (Bird and Bird, 1991). In addition to the body wall, non-striated muscles exist in the pharynx and surrounding the egg-laying apparatus and rectum.

## The excretory system

**Figure 6:**
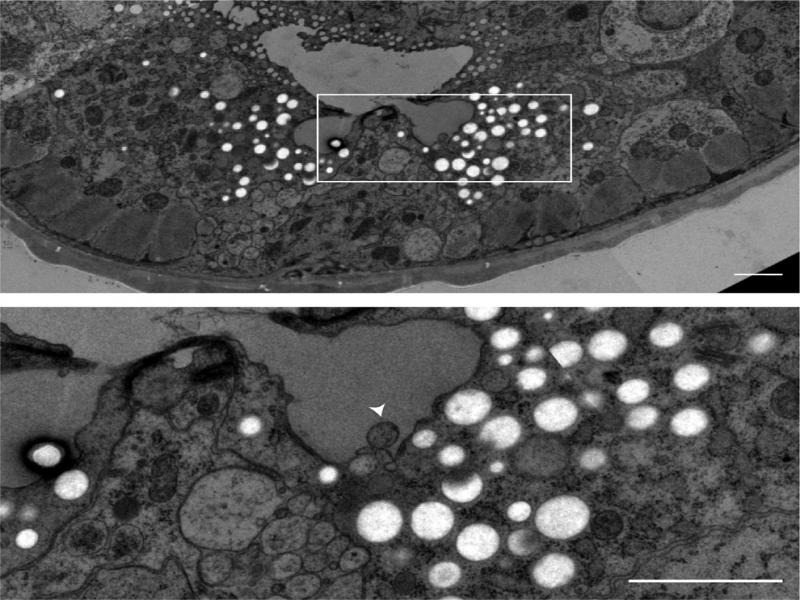
Transverse TEM micrograph through junction of excretory glands with canal cell and secretory vesicle being released in gland cell ampulla (arrowhead). Scale bars, 1 µm. Source: Ralf Sommer lab, Bumbarger13_2251.

The *P. pacificus* excretory system appears very similar to the *C. elegans* excretory system (Fig. 6). It consists of four cells—two excretory glands, a canal cell with bilaterally symmetrical longitudinal processes, and a duct cell. Similar to *C. elegans,* the *P. pacificus* CAN neuron travels alongside the canal cell processes (Carstensen et al., 2021). In *P. pacificus,* ablation of the CAN results in increased dauer formation, whereas in *C. elegans* ablation of CAN results in death (Mayer et al., 2015; White et al., 1986).

## The coloemocytes

Both *C. elegans* and *P. pacificus* adult hermaphrodites contain six large scavenger cells called coelomocytes that sit within the pseudocoelom body cavity (Jungblut and Sommer, 1998). For both species, two of the coelomocytes are generated post-embryonically (Photos et al., 2006).

## The alimentary system

**Figure 7:**
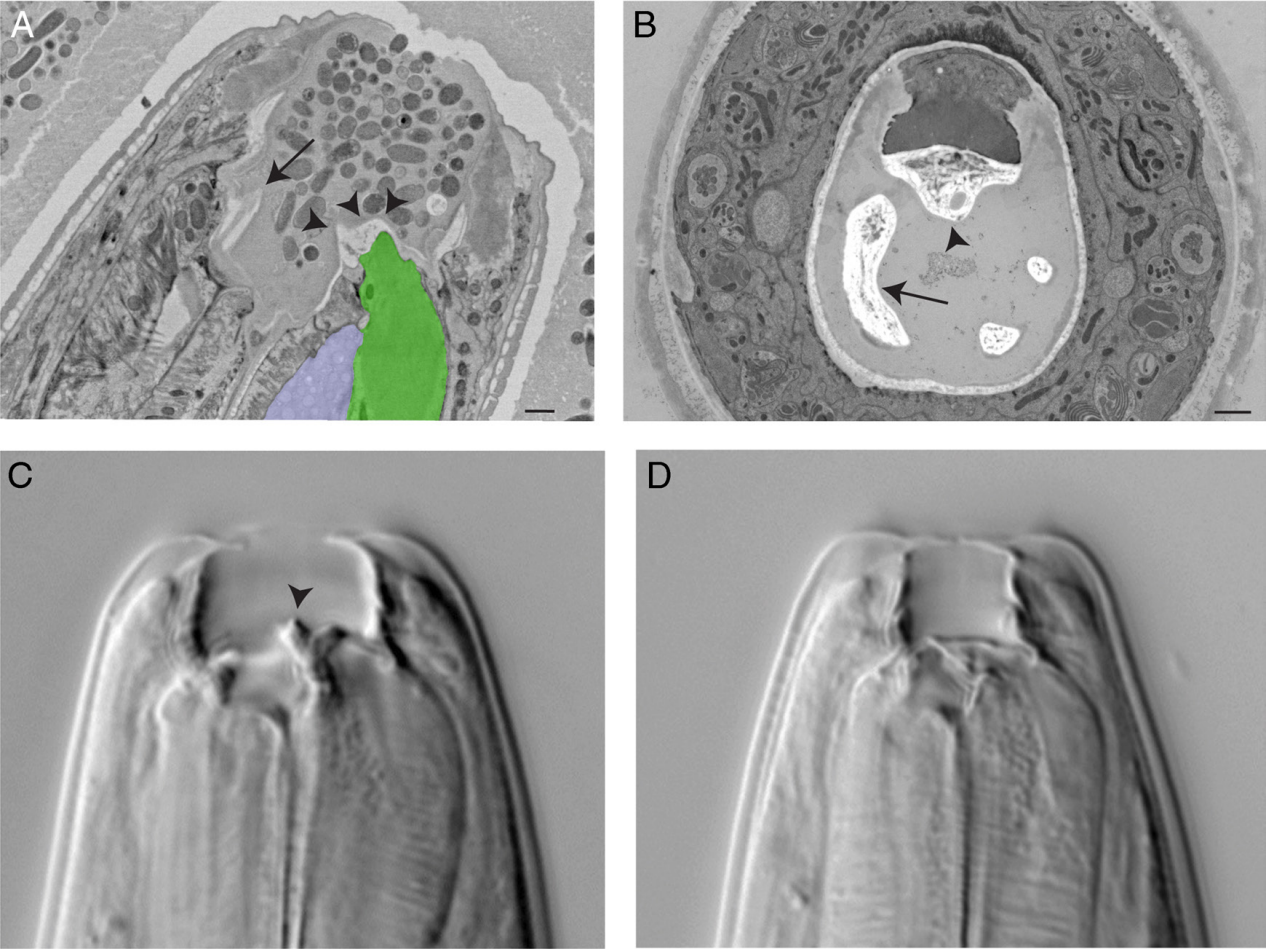
(A) Longitudinal EM through stoma of eurystomatous form with dorsal tooth (arrowheads). The dorsal tooth attaches to pharyngeal muscle (green) and contains an opening for vesicles from the dorsal pharyngeal gland (purple) to be released into the stoma. A subventral tooth (arrow) is seen on the opposite side. Scale bar, 1 µm. (B) Transverse EM micrograph through a euryostomatous stoma showing dorsal tooth (arrowhead) with lumen for dorsal pharyngeal gland secretions. A subventral tooth (arrow) is found in the subventral sector of the stoma. Scale bar, 1 µm. (Image source: Ralf Sommer Lab, Bumbarger13–154.) C,D. DIC comparison of eurystomatous (C) and stenostomatous (D) forms. The eurystomatous form contains an obvious dorsal tooth (arrowhead). (Image source: Erik Ragsdale.)

The stoma of *P. pacificus* comprises a cuticular lined cavity. Consistent with many nematodes in the Diplogastridae family, the *P. pacificus* stoma contains teeth used for predatory feeding of other nematodes (Fig. 7). The morphology of the stoma varies between two morphotypes called eurystomatous and stenostomatous that are specialized for predatory vs. microbial feeding, respectively (Serobyan et al., 2014). The eurystomatous (wide-mouthed) is characterized by a claw-like dorsal tooth and an opposing subventral tooth. The stenostomatous (narrow-mouthed) form has a less prominent triangle-shaped dorsal tooth and lacks the additional subventral tooth. The specific morphotype is determined through environmental conditions (Bento et al., 2010; Serobyan et al., 2013). *P. pacificus* dauers isolated from beetle hosts in the wild developed exclusively into eurystomatous adults (Renahan et al., 2021). The teeth of *P. pacificus* are attached to the anterior-most muscles of the pharynx, allowing for movement during feeding. Similar to *C. elegans*, the cuticle lined stoma is shed during each molt.

Posterior of the stoma, the *P. pacificus* pharynx is a muscular pump. While similar in gross morphology to the *C. elegans* pharynx, the *P. pacificus* pharynx uses a different pumping sequence to ingest food (Chiang et al., 2006). The *P. pacificus* pharynx lacks both the grinder and two gland cells found in the *C. elegans* terminal bulb; however, the three remaining gland cells of *P. pacificus* have evolved to occupy a larger fraction of the terminal bulb volume (Riebesell and Sommer, 2017).

The intestine connects to the posterior end of the pharynx. The lineage and a detailed description of the *P. pacificus* intestine are not yet available.

## The reproductive system

The first genetic analyses of *P. pacificus* focused on the molecular genetics of vulva formation (Sommer and Sternberg, 1996). Similar to *C. elegans,* the reproductive system of *P. pacificus* consists of a somatic gonad, the germ line, and the egg-laying apparatus; however, there is substantial divergence in the number of cells, overall shape, and developmental timing of the reproductive systems between *C. elegans* and *P. pacificus* (Kolotuev and Podbilewicz, 2004; Rudel et al., 2005).

Similar to *C. elegans,* the *P. pacificus* vulva is formed through a combination of cell division, migration and fusion from three ectoblastic vulval precursor P cells; however, the molecular signal controlling division differs between the species (Sommer and Sternberg, 1996). Following induction, the P cells undergo divisions to form 20 vulval cells (two less than *C. elegans*) (Kolotuev and Podbilewicz, 2004). The vulval cells migrate to form a stack of rings at the location of the vulva and undergo cell-cell fusion to form stacked toroids. The timing, sequence and number of toroids differs between *C. elegans* and *P. pacificus* (Kolotuev and Podbilewicz, 2004).

**Figure 8:**
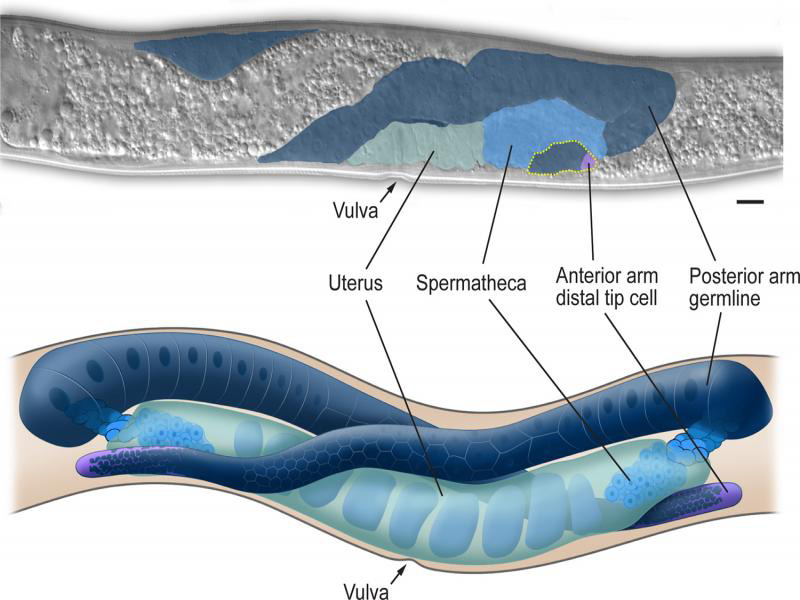
*P. pacificus* young adult hermaphrodite gonad. Unlike in *C. elegans*, the distal arms of the *P. pacificus* gonad extend back to the ventral side. Scale bar, 10 µm.

The gonad of *P. pacificus* is didelphic comprising two arms converging on a vulva positioned near the center of the body. The somatic gonad consists of a uterus, spermatheca, sheath, and distal tip cells. The germline is a syncytium connected through a central rachis. Several features of the gonad in *P. pacificus* distinguish it from that in *C. elegans* (Rudel et al., 2005). Most obviously distinct in the *P. pacificus* gonad is the pretzel shape of the gonad arms, which extend from the ventral side to the dorsal side and back again (Fig. 8). The sheath cells wrap the proximal oocytes of both species but differ in number between species (4 pairs in *P. pacificus,* 5 in *C. elegans*). While both species have sperm storage organs called spermatheca, *P. pacificus* does not contain the connective uterine-spermatheca valve cells found in *C. elegans* and differs in number of cells (Rudel et al., 2005).
